# Microbiome Signature of Endophytes in Wheat Seed Response to Wheat Dwarf Bunt Caused by Tilletia controversa Kühn

**DOI:** 10.1128/spectrum.00390-22

**Published:** 2023-01-10

**Authors:** Zhaoyu Ren, Amanda Juan Chen, Qianqian Zong, Zhenzhen Du, Qingyuan Guo, Taiguo Liu, Wanquan Chen, Li Gao

**Affiliations:** a State Key Laboratory for Biology of Plant Disease and Insect Pests, Institute of Plant Protection, Chinese Academy of Agricultural Sciences, Beijing, People’s Republic of China; b Microbiome Research Center, Moon (Guangzhou) Biotech Ltd., Guangzhou, People’s Republic of China; c Xinjiang Agricultural University, Urumqi, Xinjiang, People’s Republic of China; State Key Laboratory of Microbial Resources, Institute of Microbiology, Chinese Academy of Sciences

**Keywords:** wheat seed, *Tilletia controversa* Kühn, wheat dwarf bunt, amplicon sequencing, isolation, endophytes

## Abstract

Wheat dwarf bunt leads to the replacement of seeds with fungal galls containing millions of teliospores of the pathogen Tilletia controversa Kühn. As one of the most devastating internationally quarantined wheat diseases, wheat dwarf bunt spreads to cause distant outbreaks by seeds containing teliospores. In this study, based on a combination of amplicon sequencing and isolation approaches, we analyzed the seed microbiome signatures of endophytes between resistant and susceptible cultivars after infection with T. controversa. Among 310 bacterial species obtained only by amplicon sequencing and 51 species obtained only by isolation, we found 14 overlapping species by both methods; we detected 128 fungal species only by amplicon sequencing, 56 only by isolation, and 5 species by both methods. The results indicated that resistant uninfected cultivars hosted endophytic communities that were much more stable and beneficial to plant health than those in susceptible infected cultivars. The susceptible group showed higher diversity than the resistant group, the infected group showed more diversity than the uninfected group, and the microbial communities in seeds were related to infection or resistance to the pathogen. Some antagonistic microbes significantly suppressed the germination rate of the pathogen’s teliospores, providing clues for future studies aimed at developing strategies against wheat dwarf bunt. Collectively, this research advances the understanding of the microbial assembly of wheat seeds upon exposure to fungal pathogen (T. controversa) infection.

**IMPORTANCE** This is the first study on the microbiome signature of endophytes in wheat seed response to wheat dwarf bunt caused by Tilletia controversa Kühn. Some antagonistic microbes suppressed the germination of teliospores of the pathogen significantly, which will provide clues for future studies against wheat dwarf bunt. Collectively, this research first advances the understanding of the microbial assembly of wheat seed upon exposure to the fungal pathogen (T. controversa) infection.

## INTRODUCTION

Wheat (Triticum aestivum L.) is a key cereal crop for human food supply and one of the most widely cultivated staple food crops worldwide (1). Even though global wheat production reaches approximately 750 million metric tons, it still faces big challenges in supplying the human population, which will reach 9 billion by 2050 (2); plant diseases critically restrict crop yields worldwide (3). In particular, wheat production is severely threatened by seedborne diseases such as dwarf bunt caused by Tilletia controversa Kühn, which leads to an average yield loss of 80% and can even lead to total yield loss. As one of the most devasting internationally quarantined wheat diseases, this pathogen is the first-class quarantine target in over 30 countries. Distant epidemics of the disease can be transmitted via seeds containing teliospores, which can live in soil for up to 10 years and can still germinate successfully in a suitable environment and then infect wheat plants, ultimately replacing normal wheat seeds with fungal galls (4).

Disease prevalence may suppress pathogens and be severely affected by the assembly and composition of plant-associated microbes (5–9), and the microbial taxa of pathogen-infected and uninfected plants are significantly different (10). Highly diverse constituents of the plant microbiota have been reported in tomato (11), which reported that the rhizosphere microbiome structure alters to enable wilt resistance.

Plant seeds provide a stable environment for microbial communities (12–14). The seed microbiota can influence seed germination and seedling phenotype or drive root microbiota assembly, with the potential to promote plant growth and sustainably protect crops from pathogens (15–20). Assessing the impacts of seed treatments on microbial diversity will be one of the key factors in controlling pathogen invasion (21, 22). Romero-Severson et al. (23) isolated a strain of Bacillus safensis from surface-sterilized mung bean seeds that exhibited antibacterial activity against Escherichia coli, Xanthomonas axonopodis, and Pseudomonas syringae. Mukherjee et al. (24) isolated Pseudomonas aeruginosa BHUJPCS-7 from chickpea seeds that inhibited the growth of the pathogen Fusarium oxysporum f. sp. *ciceris*. Majumdar et al. (25) analyzed the changes in maize kernel microbiota following Aspergillus flavus infection and identified endophytes with potential antifungal/antiaflatoxin properties. Bacterial seed endophyte shapes disease resistance in rice (19). Based on the wheat seed microbiota, we know that the seedborne microbiome differs between wild and domesticated wheat species (26) and is not statistically significantly dependent on the wheat cultivar (27). The endophytic core microbiome showed a difference (20), and grain response to fumigation also showed a difference (28). Some wheat microbiomes can reduce the virulence of fungal pathogens (29). All these studies demonstrate the potential of plant seed endophytes to control plant diseases. However, reports on wheat seed microbes are affected by seedborne disease of wheat dwarf bunt are lacking.

Controlling the disease resistance of host plants is considered a promising alternative and an environmentally friendly approach (2). Microbial communities have attracted great interest in developing strategies that reshape plant microbiomes to obtain diverse conditions with antagonist agents to control plant pathogens (30). High microbial diversity could also increase competition among pathogens and microbes, which could play an important role in pathogen suppression (31, 32). The use of these microbial communities to control pathogens is the best alternative to chemical control (33). The control of wheat dwarf bunt usually depends on stirring a fungicide together with the seeds to prevent disease breakout (34). However, fungicides are not environmentally friendly. The main objective of this study was to investigate the overall composition of the microbial endophytic community of wheat seeds with T. controversa infection based on detecting naturally occurring microbial species in wheat seeds and determining their roles as antagonistic agents. Based on the analysis of the microbial communities under T. controversa attack, this study will explore the microbiome signature of endophytes in the seed to increase the understanding of endophytic community structure and supply information that will provide a promising selection and contribute to the control of wheat seedborne disease in an eco-friendly manner.

## RESULTS

### Endophytic microorganisms obtained based on isolation.

For endophytic bacteria, a total of 1,545 isolates were obtained from wheat seed, and 1,392 isolates were identified (see Table S2 in the supplemental material). Bacillus nealsonii (303 isolates, 22%), Curtobacterium plantarum (298 isolates, 21%), and Curtobacterium flaccumfaciens (105 isolates, 8%) were the main endophytic bacteria (Table S3; Fig. S1A to F). For endophytic fungi, 661 isolates from wheat seed were obtained, and 636 isolates were identified (Table S2). Aureobasidium pullulans (184 isolates, 29%) was the most frequently identified fungus, followed by *Alternaria* spp. (59 isolates, 9%) and *Didymella* spp. (48 isolates, 8%) (Table S4; Fig. S1G to N).

### Relative abundances of endophytic microorganisms.

**FIG 1:**
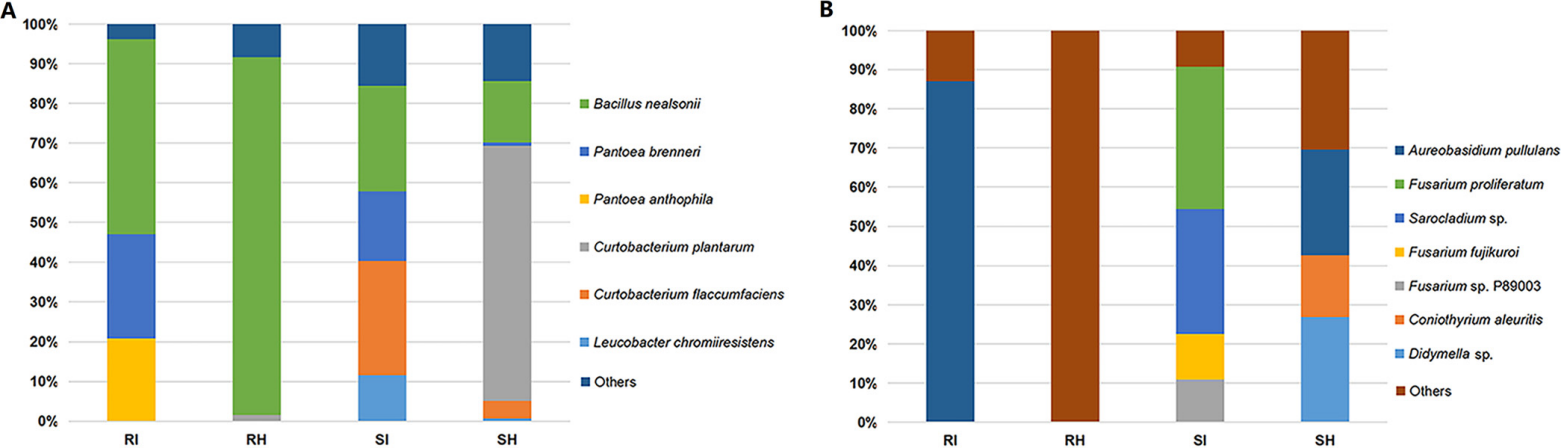
Total abundance of endophytic bacteria and fungi in different groups. (A) Total abundance of endophytic bacteria in different groups. (B) Total abundance of endophytic fungi in different groups. RI, T. controversa-infected resistant cultivar; RH, uninfected resistant cultivar; SI, T. controversa-infected susceptible cultivar; SH, uninfected susceptible cultivar.

The total relative abundances of the main endophytic microorganisms were visualized (Fig. 1; Table S5), revealing that the relative abundances of endophytic microorganisms varied among cultivars and changed after T. controversa infection. For bacteria, B. nealsonii was dominant in resistant cultivars but lower in relative abundance in susceptible cultivars. With the statistical tests, the relative abundance of C. plantarum was higher in the uninfected groups (uninfected resistant cultivar [RH] and uninfected susceptible cultivar [SH]) than in the other treatments, and the relative abundance of Pantoea brenneri was elevated after T. controversa infection (Fig. 1A). For the fungi, A. pullulans was dominant in the uninfected susceptible cultivar and the T. controversa-infected resistant cultivar. Fusarium proliferatum, *Sarocladium* sp., and Fusarium fujikuroi were dominant in the susceptible infected group (Fig. 1B).

### Amplicon sequencing and bioinformatics pipeline.

Rarefaction curves of microbial communities approached asymptotes, indicating the sufficiency of our sequence depth for bacteria (Fig. S2A) and fungi (Fig. S2B). For infected and uninfected resistant and susceptible cultivars, some sequences were randomly selected to construct amplicon sequence variants (ASVs). As the sequence depth increased, the number of ASVs increased rapidly and leveled off, which indicated that additional sequence depth would not increase the number of ASVs. This signifies that the sequencing depth meets the requirements.

### Diversity analysis of endophytic community composition.

Comparative analyses of Shannon diversity were performed after bacterial and fungal sequences were normalized (Fig. S2C and D). By the Wilcoxon rank-sum test (*P < *0.05), the Shannon diversity analysis of 16S data showed that the diversity decreased after T. controversa infection in both susceptible and resistant cultivars, and the Shannon diversity of the infected susceptible cultivar (SI) was the lowest (Fig. S2C). Shannon diversity analysis of internal transcribed spacer (ITS) data showed that the diversity was reduced after T. controversa infection in the resistant cultivar but increased after T. controversa infection in the susceptible cultivar (Fig. S2D). This result suggested that infection with T. controversa decreased the diversity of infection resistance and susceptibility for bacteria, while it caused higher diversity in susceptible infected cultivars than in resistant infected cultivars.

**FIG 2:**
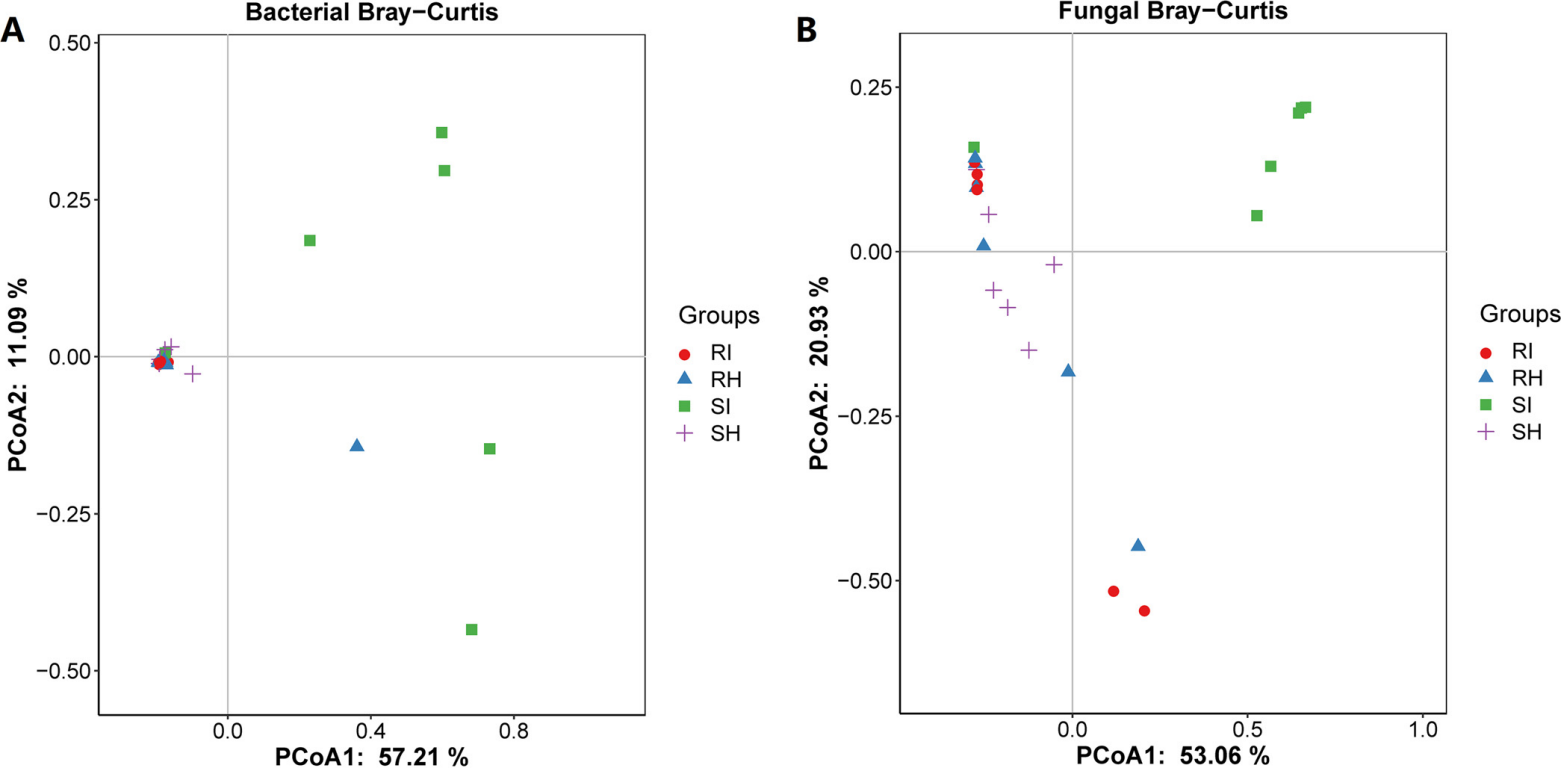
Principal-coordinate analysis (PCoA) of microbial communities based on Bray-Curtis analysis. (A) Bray-Curtis analysis of bacterial communities. (B) Bray-Curtis analysis of fungal communities. RI, T. controversa-infected resistant cultivar; RH, uninfected resistant cultivar; SI, T. controversa-infected susceptible cultivar; SH, uninfected susceptible cultivar.

Principal-coordinate analysis (PCoA) was used to compare the effects of T. controversa infection on the microbial community structures of different wheat cultivars, and the principal components indicated variations of 57.21% and 53.06% for bacterial and fungal communities, respectively. The bacterial communities of the susceptible cultivar infected by T. controversa (SI) were mainly distributed in the first and fourth quadrants (Fig. 2A), while the infected resistant cultivar (RI) and SH were clustered together. The fungal communities of the SI were mainly found in the first quadrant and separated from the other groups (Fig. 2B). The Bray-Curtis analyses of bacteria and fungi were performed with permutational multivariate analysis of variance (PERMANOVA), which showed significant differences based on *P* values of <0.05 (Table S6) (35). Collectively, PCoA showed that T. controversa infection significantly changed the endophytic community structure of the susceptible cultivars.

### Cultivars and infection-associated seed endophytes.

**FIG 3:**
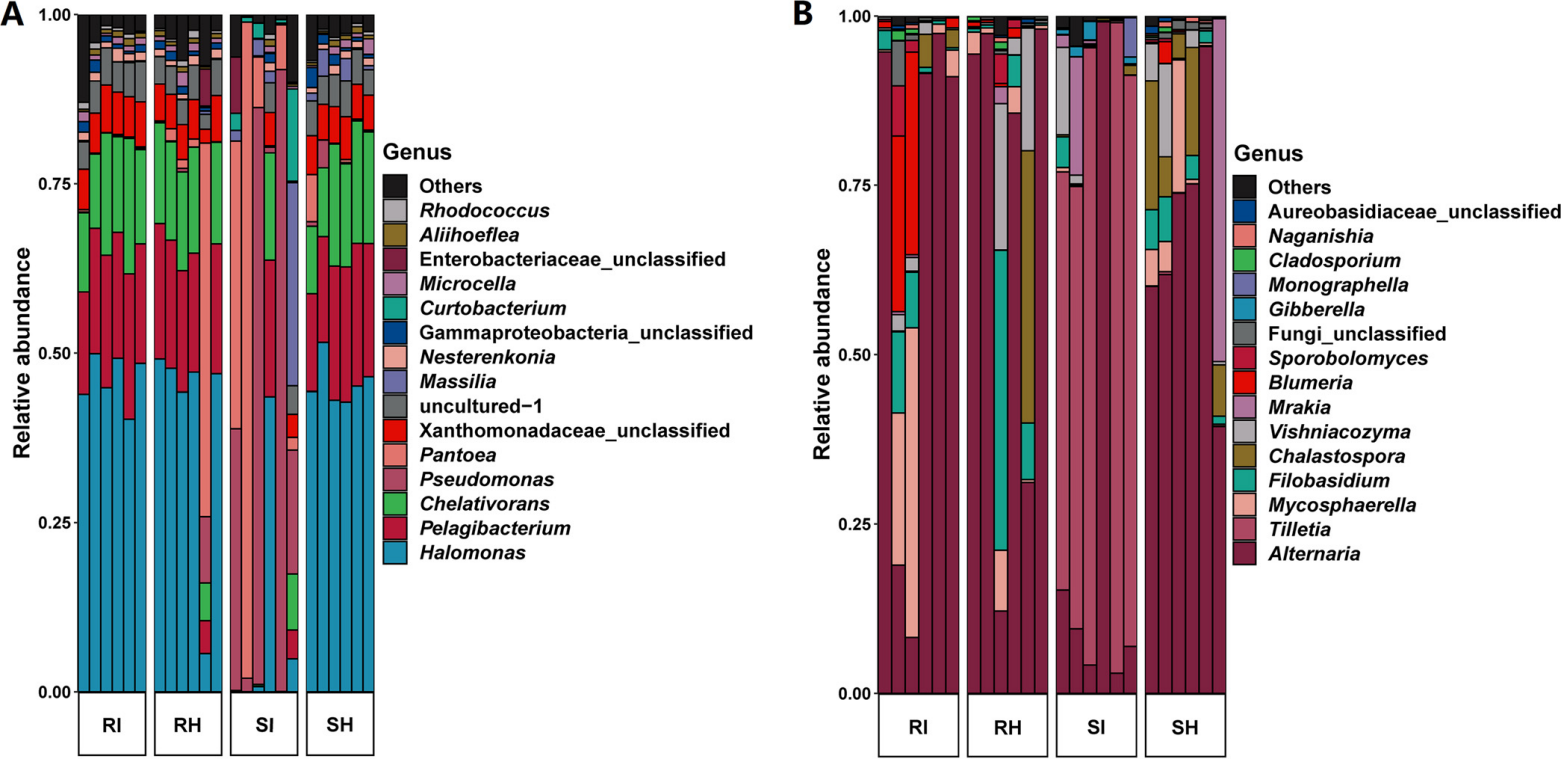
The relative abundances of bacteria and fungi at the genus level by amplicon sequencing. (A) The relative abundances of bacterial taxa in T. controversa-infected and uninfected resistant and susceptible wheat samples. (B) The relative abundances of fungal taxa in T. controversa-infected and uninfected resistant and susceptible wheat samples. RI, T. controversa-infected resistant cultivar; RH, uninfected resistant cultivar; SI, T. controversa-infected susceptible cultivar; SH, uninfected susceptible cultivar.

We assessed microbial taxa associated with T. controversa-infected and uninfected samples. As shown in Table S7, 155 bacterial genera and 81 fungal genera were found among all the samples by amplicon sequencing, and the phyla *Proteobacteria* and Ascomycota were dominant for the bacterial and fungal communities, respectively. As shown in Fig. 3A, amplicon sequencing revealed that the dominant bacterial genera included *Halomonas*, *Pelagibacterium*, *Chelativorans*, Pseudomonas, *Pantoea*, *Xanthomonadaceae*-unclassified, *Massilia*, and *Nesterenkonia*. Additionally, *Halomonas*, *Pelagibacterium*, and *Chelativorans* were dominant in the infected RI group and the SH group, while Pseudomonas was dominant in 3 of the 6 samples of the SI group, and *Nesterenkonia* was dominant in 2 of the 6 samples of SI. Amplicon sequencing showed that the dominant fungal genera included *Alternaria*, *Tilletia*, *Mycosphaerella*, *Filobasidium*, *Chalastospora*, *Vishniacozyma*, *Mrakia*, *Blumeria*, and *Sporobolomyces* (Fig. 3B). Additionally, *Alternaria* was dominant in the SH, while *Tilletia* was dominant in 5 of 6 samples of the T. controversa*-* infected SI group.

**FIG 4:**
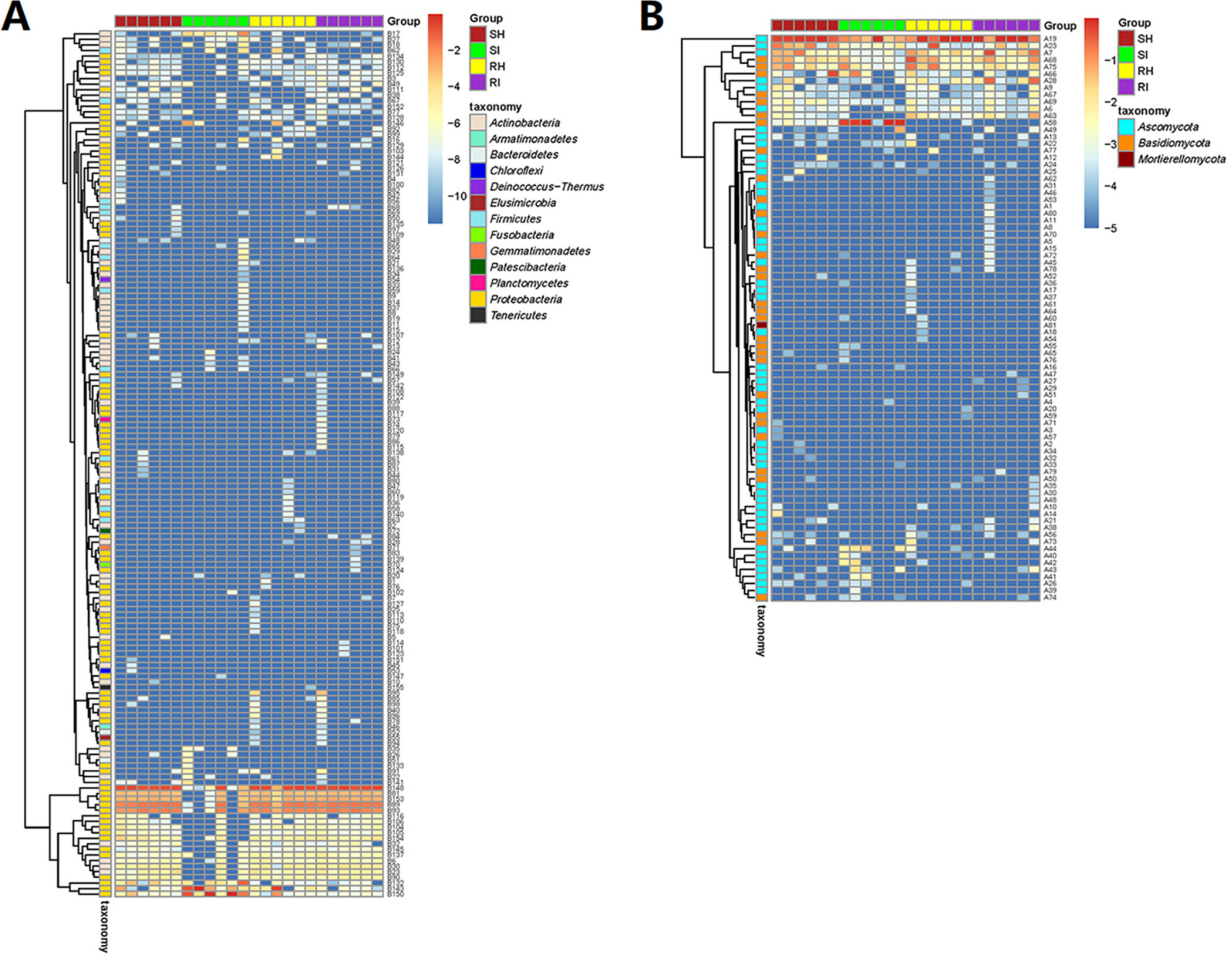
Heatmaps of enriched bacterial and fungal genera by amplicon sequencing. (A) Heatmap of bacterial genera in all groups. (B) Heatmap of fungal genera in all groups. The dendrogram shows the clustering tree. The box was colored based on the relative abundance data. The red color shows a higher abundance of genus, and the blue color shows a lower abundance. The number on the right corresponds to Table S7 in the supplemental material. RI, T. controversa-infected resistant cultivar; RH, uninfected resistant cultivar; SI, T. controversa-infected susceptible cultivar; SH, uninfected susceptible cultivar.

Heatmap analysis was conducted to show the distribution of all microbes at the genus level (Fig. 4). Based on the information in Table S7, for the bacterial analysis shown in Fig. 4A, *Curtobacterium* (Table S7, B17) and Pseudomonas (Table S7, B150) were increased in the infected susceptible cultivar, while *Chryseobacterium* was decreased in the infected susceptible cultivar. In Fig. 4B, fungal analysis showed that *Alternaria* was decreased in the infected susceptible cultivar. Overall, the susceptible infected group showed different trends from the other groups (Fig. 4A and B).

Based on the *P* values for the fold changes in relative abundance, we constructed a rectangular coordinate system (Fig. S3). For bacteria, we found that Pseudomonas and *Curtobacterium* were enriched in the SI group, while *Halomonas* and *Xanthomonadaceae* were enriched in the SH group (Fig. S3A). There were no significant differences between infected and uninfected resistant cultivars (RH, RI) as well as the uninfected susceptible and resistant cultivars (SH, RH) (Fig. S3B and C). *Pantoea*, Pseudomonas, and *Curtobacterium* were enriched in the SI group, while *Xanthomonadaceae*, *Halomonas*, and *Corynebacterium* were enriched in the RI group (Fig. S3D). For fungi, we found that *Mycosphaerella* was enriched in the SI group (Fig. S3E). There were no significant differences between the RH and RI groups (Fig. S3F). The relative abundance of *Tilletia* was higher in the SH group than in the RH group (Fig. S3G) and higher in the SI group than in the RI group (Fig. S3H). In summary, there were no microorganisms that were significantly different before and after infection in the resistant group, but some microorganisms showed significant differences before and after infection in the susceptible group.

### Cross-kingdom connectivity of endophytic microbiota.

**FIG 5:**
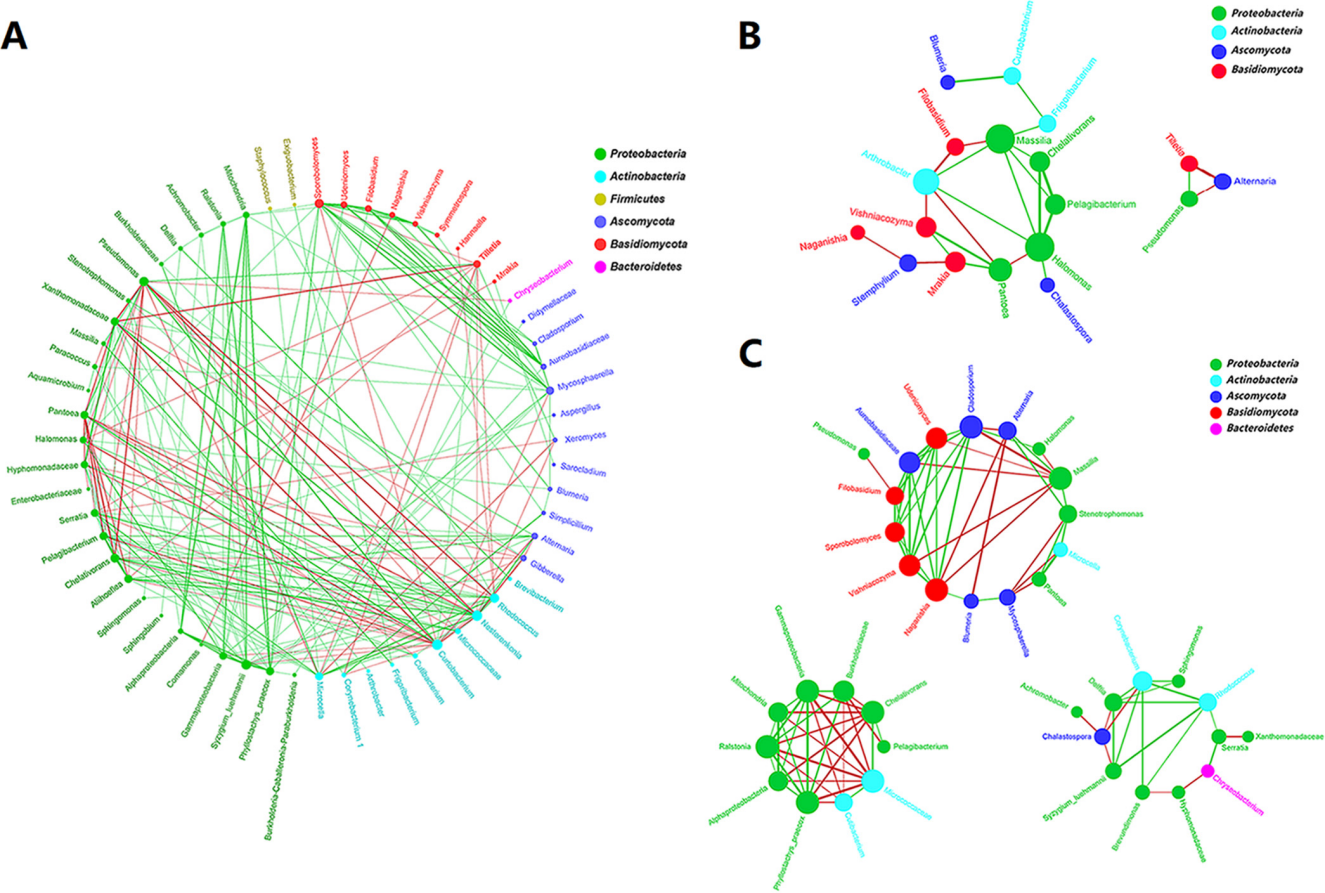
Visualization of microbial community co-occurrence network properties between uninfected and infected groups. (A) Potential keystone taxa based on bacterial and fungal network analysis of all groups. (B) Co-occurrence networks of diseased groups. (C) Co-occurrence networks of uninfected groups. Nodes represent ASVs and are colored by bacterial and fungal phyla.

Separate co-occurrence networks were constructed for T. controversa-infected RI and SI cultivars to explore differences in co-occurrence patterns. The topological properties of the co-occurring networks are shown in Table S8. In Fig. 5, each point is colored according to the phylum, and the size of each point is positively related to the number of connecting lines. After combining the bacterial and fungal relative abundance data, a co-occurrence network diagram was obtained for all samples. *Xanthomonadaceae*, *Halomonas*, *Aliihoeflea*, *Microcella*, *Corynebacterium*, *Nesterenkonia*, and *Rhodococcus* were negatively correlated with *Tilletia* (Fig. 5A), which is also indicated in Fig. 3A. *Xanthomonadaceae*, *Halomonas*, and *Rhodococcus* were reduced in infected compared to uninfected groups of susceptible cultivars. We also found that *Alternaria* showed a negative correlation with *Tilletia*, while Pseudomonas showed a positive correlation with *Tilletia* (Fig. 5B). *Bacteroidetes* occurred in the RI group, suggesting that *Bacteroidetes* may contribute to the disease resistance of wheat (Fig. 5C). The network for the RI group was characterized by more links between nodes, a greater clustering coefficient, and longer average path lengths than that for the SI (Fig. 5B and C), indicating that the close associations between endophytes were responsible for the resistance of cultivars.

### Overlapping endophytic microorganisms from isolation and amplification sequencing.

At the species level, 51 bacterial species were isolated (Table S3), 310 bacterial species were obtained by amplicon sequencing (Table S9), and 14 were shared between the two methods (Fig. S2E), including Bacillus anthracis, Pseudomonas graminis, C. flaccumfaciens, Erwinia tasmaniensis, Leucobacter alluvii, P. brenneri, Pseudomonas azotoformans, Pantoea agglomerans, Sanguibacter inulinus, Shigella flexneri, Massilia suwonensis, Brevundimonas intermedia, and Pseudomonas libanensis. For fungi, 56 fungal species were identified from the culture media (Table S4), 128 fungal species were obtained by amplicon sequencing (Table S9), and 5 of them overlapped (Fig. S2F), including Fusarium solani, Aspergillus penicillioides, Sarocladium strictum, Fusarium equiseti, and Cladosporium australis.

### Antagonists against the germination of teliospores of T. controversa.

**FIG 6:**
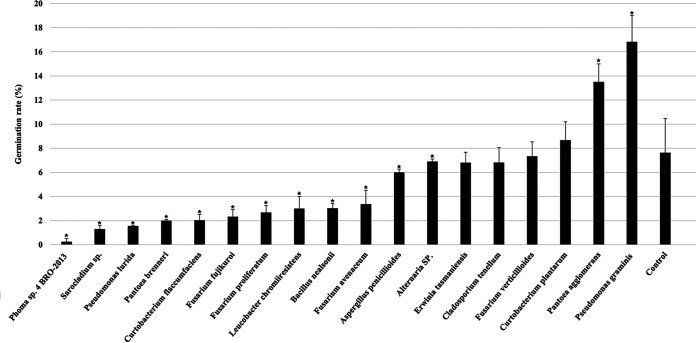
Effects of potential antagonist isolates on the germination of T. controversa teliospores. *, *P* < 0.05. RI, T. controversa-infected resistant cultivar; RH, uninfected resistant cultivar; SI, T. controversa-infected susceptible cultivar; SH, uninfected susceptible cultivar.

Based on the isolation method, together with considering relative abundance (Table S10), potential antagonistic microorganisms were chosen. These microorganisms were evaluated for their effects on the germination of teliospores of T. controversa. The germination rates indicated that *Phoma* sp. strain 4 BRO-2013, *Sarocladium* sp., Pseudomonas lurida, P. brenneri, C. flaccumfaciens, F. fujikuroi, F. proliferatum, Leucobacter chromiiresistens, B. nealsonii, Fusarium avenaceum, A. penicillioides, and *Alternaria* sp. inhibited the germination of teliospores significantly, while E. tasmaniensis, Cladosporium tenellum, and Fusarium verticillioides did not show significant difference (Fig. 6, Fig. S4, and Table S11).

## DISCUSSION

A large amount of the microbiome lives in various plant tissues and is believed to play a great role in plant fitness by resisting biotic and abiotic factors (36). To the best of our knowledge, this study is the first to combine amplicon sequencing and culture methods to analyze the changes in the wheat seed microbiomes of resistant and susceptible cultivars in response to the pathogen T. controversa, which could increase our understanding of the role of the microbiome as an antagonist against seedborne wheat dwarf bunt disease.

In our study, amplicon sequencing (310 bacterial species in 155 genera and 128 fungal species in 81 genera) and microbial isolation (66 bacterial species in 29 genera and 52 fungal species in 23 genera) approaches were carried out to identify endophytes in wheat seeds; 14 bacterial species and 5 fungal species overlapped in both methods, suggesting a complementary relationship between culture-dependent and culture-independent methods (37), and the combination of the two approaches was able to obtain more microorganisms.

High microbial diversity is involved in plant defense and can contribute to disease suppression (38–41). In this study, PCoA indicated that the microbial communities changed significantly after T. controversa infection in the susceptible cultivar (Fig. 2). Shannon diversity analysis showed that the bacterial diversity was lower in the susceptible cultivar than in the resistant cultivar (RI) after T. controversa infection (see Fig. S2C in the supplemental material), which indicates that bacterial diversity contributes to resistance to T. controversa.

Infection with pathogens changed the relative abundance of host endophytes (7, 42–44). In this study, for isolated bacterial communities (Fig. 1A), the relative abundance of P. brenneri increased after T. controversa infection. *Pantoea* spp. have been reported to secrete antimicrobial agents and have been developed into commercial biocontrol products to help control fire blight in apple and pear trees (45–47); thus, the increase in relative abundance indicates that P. brenneri is involved in the process of plants resisting T. controversa. For the isolated fungal communities shown in Figure 1B, the relative abundances of A. pullulans, *Didymella* sp., and *Coniothyrium aleuritis* decreased after T. controversa infection in the susceptible cultivar, while the relative abundances of F. proliferatum, F. fujikuroi, and Fusarium sp. strain P89003 were increased after T. controversa infection in the susceptible cultivar. Previous reports mentioned that Stagonospora nodorum-infected plants were associated with several other infected pathogens (48); similarly, in our study, we found that the infection of T. controversa in susceptible cultivars was also related to F. proliferatum, F. fujikuroi, and Fusarium sp. P89003 (Fig. 1B).

An increased abundance of Pseudomonas spp. has been found to significantly decrease pathogen severity and disease incidence by interfering with pathogens (10, 49). Heatmap analysis showed that Pseudomonas was increased after T. controversa infection in the susceptible cultivar (Fig. 4A), indicating that Pseudomonas was involved in the process of resisting T. controversa in the susceptible cultivar. Poudel et al. (50) revealed that co-occurrence network analysis of plant microbiomes can provide new perspectives for enhancing disease management and identifying candidate microbes affecting plant health. The results of our co-occurrence network analysis highlighted *Xanthomonadaceae*, *Halomonas*, *Aliihoeflea*, *Microcella*, *Corynebacterium*, *Nesterenkonia*, and *Rhodococcus*, suggesting that these genera exerted inhibitory effects on *Tilletia* (Fig. 5A), and *Bacteroidetes* was only found in resistant infected cultivars (Fig. 5C). *Bacteroidetes* spp. have been reported to have high efficiency against fungal pathogens (51). *Corynebacterium* spp. have exhibited ability to reduce the disease severity of northern leaf blight caused by Exserohilum turcicum (52). *Halomonas* spp. and *Nesterenkonia* spp. have been reported to have antifungal potential (53). *Rhodococcus* spp. was reported as a potential biocontrol agent in suppressing black rot disease, Rhizobium rhizogenes, Fusarium moniliforme, F. oxysporum, and Botryosphaeria dothidea (54–56). All these studies indicate the potential of these endophytes as antagonists of T. controversa.

Manipulating plant endophytes can improve plant disease resistance (18). In our study, we isolated some potential antagonistic microorganisms that inhibited the germination of T. controversa, such as *Phoma* sp. 4 BRO-2013, *Sarocladium* sp., P. lurida, P. brenneri, C. flaccumfaciens, F. fujikuroi, F. proliferatum, L. chromiiresistens, B. nealsonii, F. avenaceum, A. penicillioides, and *Alternaria* sp. (Fig. 6). In particular, the *Sarocladium* sp. significantly inhibited the germination rate of T. controversa and was previously reported to inhibit the growth of phytopathogenic fungi, such as A. flavus and F. verticillioides (57). P. lurida can protect Caenorhabditis elegans against pathogens such as Bacillus thuringiensis (58). C. flaccumfaciens has been reported against Erwinia amylovora (59). A detailed program of study is needed on the microbial potential of antagonistic T. controversa in the future.

In conclusion, these results suggest that T. controversa prevalence was lower in resistant cultivars in which higher microbial diversity was featured, and specific promising antagonists potentially contributed to their pathogen suppression ability.

## MATERIALS AND METHODS

### Seed treatment, plant culture, and pathogen inoculation.

For seed surface disinfection, 1.25 g of seeds was soaked in 75% ethanol for 2 min, washed with 0.01% Tween 80 in an ultrasonic cleaner, and rinsed with double-distilled water (ddH_2_O) 3 times. Then, the wheat seeds were sterilized with 5% sodium hypochlorite for 5 min, rinsed with ddH_2_O 3 times, and treated with 75% ethanol for 3 min (60). Finally, the wheat seeds were rinsed with ddH_2_O 3 times, and 200 μL of the 2nd rinse water was added into lysogeny broth medium and placed in a 28°C incubator for 7 days and a 37°C incubator for 3 days for disinfection confirmation (61). Afterward, 1.25 g of the sample was divided into 1 g for the isolation of endophytes and 0.25 g for the extraction of the amplicon sequencing of endophytes. For plant culture, a total of 24 wheat cultivars were selected from the Institute of Plant Protection, Chinese Academy of Agricultural Sciences, China, of which 12 wheat cultivars were resistant to T. controversa, and 12 were susceptible cultivars.

Disinfected seeds were germinated in sterile moist muslin cloth for 1 month, planted in pots (mixed with organic matter and soil at a ratio of 1:2), and placed into growth chambers (LT-36VL; Percival Scientific, USA) under 24 h of light and 60% relative humidity. In the early stage of seedling growth, the temperature was adjusted to 15°C, and then the temperature was gradually increased in the later stage. Finally, the temperature was adjusted to 20°C in the boot stage. Spores of T. controversa were inoculated into the root zone of wheat plants, and this process was repeated 5 times at 1-day intervals. Plants that received ddH_2_O inoculation were used as controls. T. controversa was kindly provided by Blair Goates, the United States Department of Agriculture (USDA), Agricultural Research Service (ARS), United States. Seven replicates were used for every treatment (seven spikes each were taken from the inoculated and uninoculated wheat of each cultivar). Information about the wheat cultivars used in this experiment is listed in Table 1.

**TABLE 1:** List of the varieties used in this study

Variety name	Treatment	Groups	Sample ID
Resistant cultivar 1	Infected	RI	S1
Resistant cultivar 2	Noninfected	RH	S2
Resistant cultivar 3	Infected	RI	S3
Resistant cultivar 4	Noninfected	RH	S4
Resistant cultivar 5	Infected	RI	S5
Resistant cultivar 6	Noninfected	RH	S6
Resistant cultivar 7	Infected	RI	S7
Resistant cultivar 8	Noninfected	RH	S8
Resistant cultivar 9	Infected	RI	S9
Resistant cultivar 10	Noninfected	RH	S10
Resistant cultivar 11	Infected	RI	S11
Resistant cultivar 12	Noninfected	RH	S12
Susceptible cultivar 1	Infected	SI	S13
Susceptible cultivar 2	Noninfected	SH	S14
Susceptible cultivar 3	Infected	SI	S15
Susceptible cultivar 4	Noninfected	SH	S16
Susceptible cultivar 5	Infected	SI	S17
Susceptible cultivar 6	Noninfected	SH	S18
Susceptible cultivar 7	Infected	SI	S19
Susceptible cultivar 8	Noninfected	SH	S20
Susceptible cultivar 9	Infected	SI	S21
Susceptible cultivar 10	Noninfected	SH	S22
Susceptible cultivar 11	Infected	SI	S23
Susceptible cultivar 12	Noninfected	SH	S24

### Endophytic microorganism detection based on isolation.

A sterile mixing cup (FastPrep-96 sample preparation instrument [MP Biomedicals, USA]) was used to crush the surface-disinfected sample, 9 mL of 0.01% Tween 80 was added, and the sample was vortexed at 1,500 rpm for 1 min and sonicated in an ultrasonic cleaner for 3 min to obtain the first sample diluent. One milliliter from the first sample diluent was added to 9 mL of 0.01% Tween 80 and vortexed to obtain the second sample diluent, and this process was repeated for the third sample diluent. One hundred microliters of the first, second, and third sample diluents was cultured on the bacterial isolation medium separately, and 100 μL of the first and second sample diluents was cultured on the fungal isolation medium separately (61–63).

To isolate as many endophytes as possible, a variety of common media were used to cultivate microorganisms (see Table S1 in the supplemental material). Lysogeny broth (LB) medium, 25% nutrient agar medium (NA), Reasoner's 2A agar (R2A) medium, rice medium, tryptone soya agar (TSA) medium, and tap water yeast extract (TWYE) medium were used for bacterial isolation. Corn meal agar (CMA) medium, dichloran glycerol (DG18) medium, malt extract agar (MEA) medium, potato dextrose agar (PDA) medium, 25% PDA medium, resorbable blasting (RBM) medium, rice medium, and 8-vegetable juice (V8) medium were used for fungal isolation.

Bacteria were incubated at 37°C for 3 days, and fungi were cultured at 28°C for 7 days. The CFU with different phenotypes from each culture medium were transferred with sterile toothpicks into separate tubes each with 1 mL liquid medium (LB/R2A medium for bacteria and MEA/DG18 medium for fungi) and incubated for 1 day at 37°C and 200 rpm and 28°C and 180 rpm, respectively. Ten microliters of each bacterial or fungal liquid was added to 10 μL of lysis solution (8 mL EDTA, 80 mL NaH_2_PO_4_, 40 mL glycerol, ddH_2_O added to 800 mL), centrifuged at 3,000 rpm for 1 min, lysed at 95°C for 10 min, cooled to 60°C, and stored at −20°C for 15 min. Each supernatant was taken for PCR amplification. Each total reaction system volume was 27 μL and included 12 μL of 2× Ex Taq master mix (Qiagen, Germany), 2 μL of template DNA, 0.6 μL of forward primer (10 μM), 0.6 μL of reverse primer (10 μM), 1 μL dimethyl sulfoxide (DMSO), and 10.8 μL of ddH_2_O. The primer sequences for bacteria were 27F (5′-AGAGTTTGATCMTGGCTCAG-3′) and 1492R (5′-TACGGYTACCTTGTTACGACTT-3′), and those for fungi were ITS4 (5′-TCCTCCGCTTATTGATATGC-3′) and ITS5 (5′-GGAAGTAAAAGTCGTAACAAGG-3′). The 16S and ITS PCR amplification conditions were as follows: predenaturation at 94°C for 4 min; followed by 35 cycles of amplification with denaturation at 94°C for 40 s, annealing at 55°C for 40 s, and extension at 72°C for 60 s; and a final extension at 72°C for 10 min.

### Endophytic microorganism detection based on amplicon sequencing.

The FastDNA spin kit for soil (MP Biomedicals, Santa Ana, CA, USA) was used to extract DNA from 0.25-g grain samples by following the manufacturer’s instructions. DNA was used for concentration detection with a NanoDrop 3300 system (Biotech, USA). The purified DNA was sent to Shanghai Personal Biotechnology Co., Ltd., China, for MiSeq sequencing. PCR amplification of fungal ITS sequences employed the fungal primers ITS1 (5′-CTTGGTCATTTAGAGGAAGTAA-3′) and ITS2 (5′-GCTGCGTTCTTCATCGATGC-3′). For bacteria, the primers were 799F (5′-AACMGGATTAGATACCCKG-3′) and 1193R (5′-ACGTCATCCCCACCTTCC-3′). Sequence amplification was conducted on an Illumina MiSeq PE250 platform. Raw paired-end sequence reads were filtered with FASTQ (v.0.19.7) (parameter, -b 250 -B 250), which performed adapter trimming, quality profiling, and read filtering (64). High-quality reads were further analyzed using the Quantitative Insights into Microbial Ecology (QIIME2) (parameter, –type SampleData[SequencesWithQuality]) (65) bioinformatics pipeline. DADA2 qiime dada2 denoise-single (parameter, -p-trim-left 0 –p-trunc-len 0) was utilized to generate amplicon sequence variants (ASVs) by denoising and removing chimeric and short reads (66). Furthermore, the UNITE v8_99 ITS gene database (67) and the SILVA v132_99 16S rRNA gene database (68) were used to train a naive Bayesian classifier (69) to classify ASVs taxonomically. The biodiversity of the samples, including alpha and beta diversity, was calculated by using the q2-diversity plugin. The abundance of genus showed with heatmap and heatmaps were generated with pheatmap v1.0.12 (70). Significantly different genera were mined by calculating the fold changes and *P* values using the Wilcoxon rank-sum test and then visualized with a volcano plot using a ggplot2 package (71). Spearman coefficients of correlation between species were calculated based on the relative abundance at the genus level, and Cytoscape was used to construct the network with thresholds of Spearman correlation of >0.3 and *P* value of <0.01 (72).

### Suppression of the germination rates of the teliospores of T. controversa by potential antagonist isolates.

For teliospore surface disinfection, the teliospores were sterilized with 0.25% NaClO for 1 min and then washed 3 times with sterile ddH_2_O (73). The concentration was adjusted to 10^5^ teliospores/mL by a hemocytometer (Jingchen, China), and then 200 mL of suspension was added to the soil-agar medium (2%) (74). Media were maintained with a 24-h light cycle at 4°C in an incubator (MLR-352H; Panasonic, USA) for 20 days. Then, each plate was divided into two equal areas: half of the plate was treated with antagonist isolate (100 μL, 10^3^ CFU/mL), and the same volume of sterile ddH_2_O was added to the other half for the control and then incubated at 4°C for 10 days. The procedure was repeated for each potential antagonist isolate for 7 biological replicates. The germination rates of T. controversa (number of teliospores germinated in the visual field/number of total teliospores in the visual field [at least 100 teliospores] × 100%) were then calculated with each antagonist cocultivation under a microscope (Leica S6D; Germany), and analysis of variance (ANOVA) was conducted on the germination rates using SPSS 17.0 and Excel (2010).

### Data availability.

All raw sequencing data have been submitted to the NCBI Sequence Read Archive (SRA) database under the accession numbers SRX14097140 to SRX14097163 (https://www.ncbi.nlm.nih.gov/sra?linkname=bioproject_sra_all&from_uid=804347).
