# Supplementary material for: Microbiome Signature of Endophytes in Wheat Seed Response to Wheat Dwarf Bunt Caused by Tilletia controversa Kühn
Source: Microbiol Spectr. 2023 Jan 10;11(1):e00390-22. doi: 10.1128/spectrum.00390-22 (PMC9927297; doi:10.1128/spectrum.00390-22)
Supplement: Supplemental file 1 — Supplemental material. Download spectrum.00390-22-s0001.pdf, PDF file, 3.1 MB [file spectrum.00390-22-s0001.pdf]

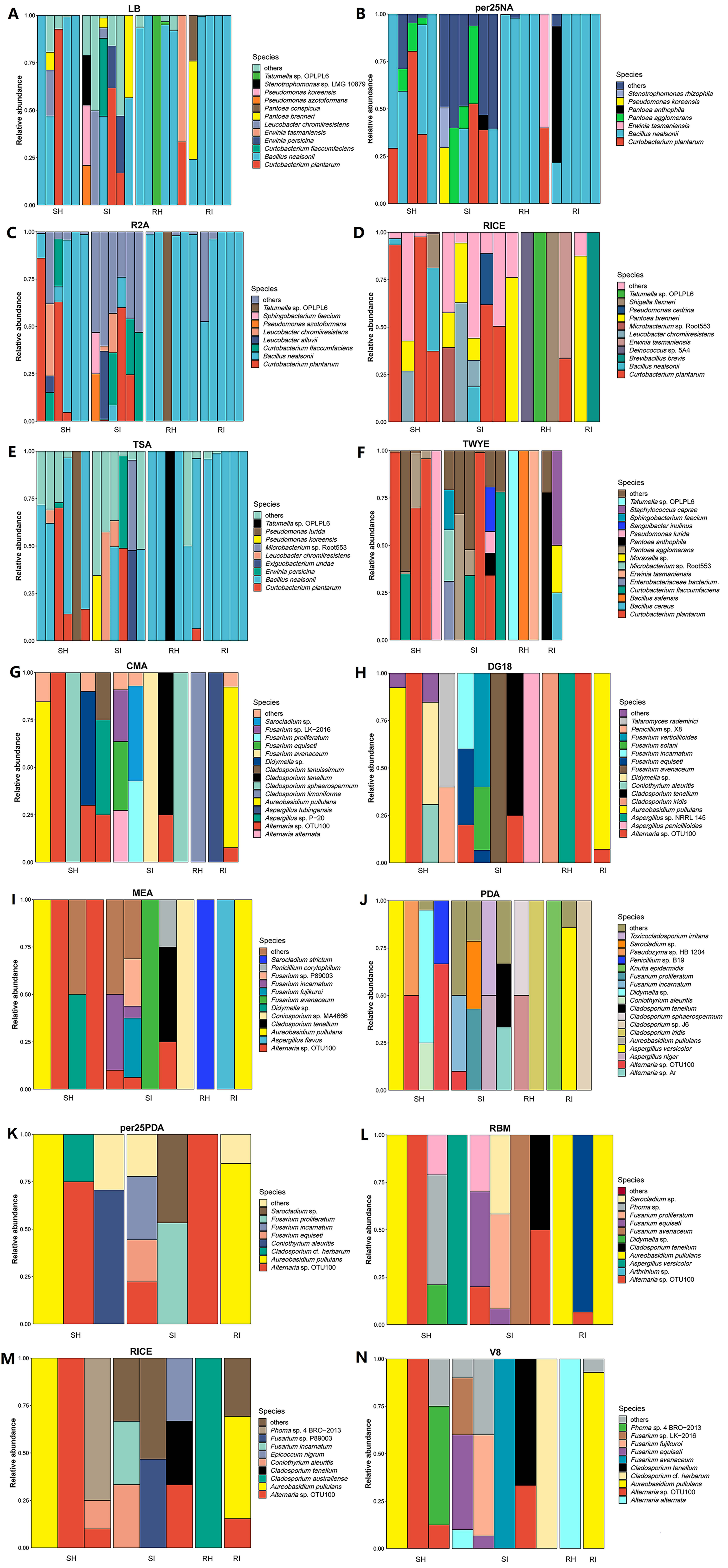

Fig. S1. Bar plot of bacteria and fungal cultured in different media. Bacteria (**A**) Cultured in LB, (**B**) Cultured in 25% PDA, (**C**) Cultured in R2A, (**D**) Cultured in RICE, (**E**) Cultured in TSA and (**F**) Cultured in TWYE. Fungi (**G**) Cultured in CMA, (**H**) Cultured in DG18, (**I**) Cultured in MEA, (**J**) Cultured in PDA, (**K**) Cultured in 25% PDA, (**L**) Cultured in RBM, (**M**) Cultured in RICE and (**N**) Cultured in VS. RI: *T. controversa*-infected resistant cultivar, RH: uninfected resistant cultivar, SI: *T. controversa*-infected susceptible cultivar, SH: uninfected susceptible cultivar.

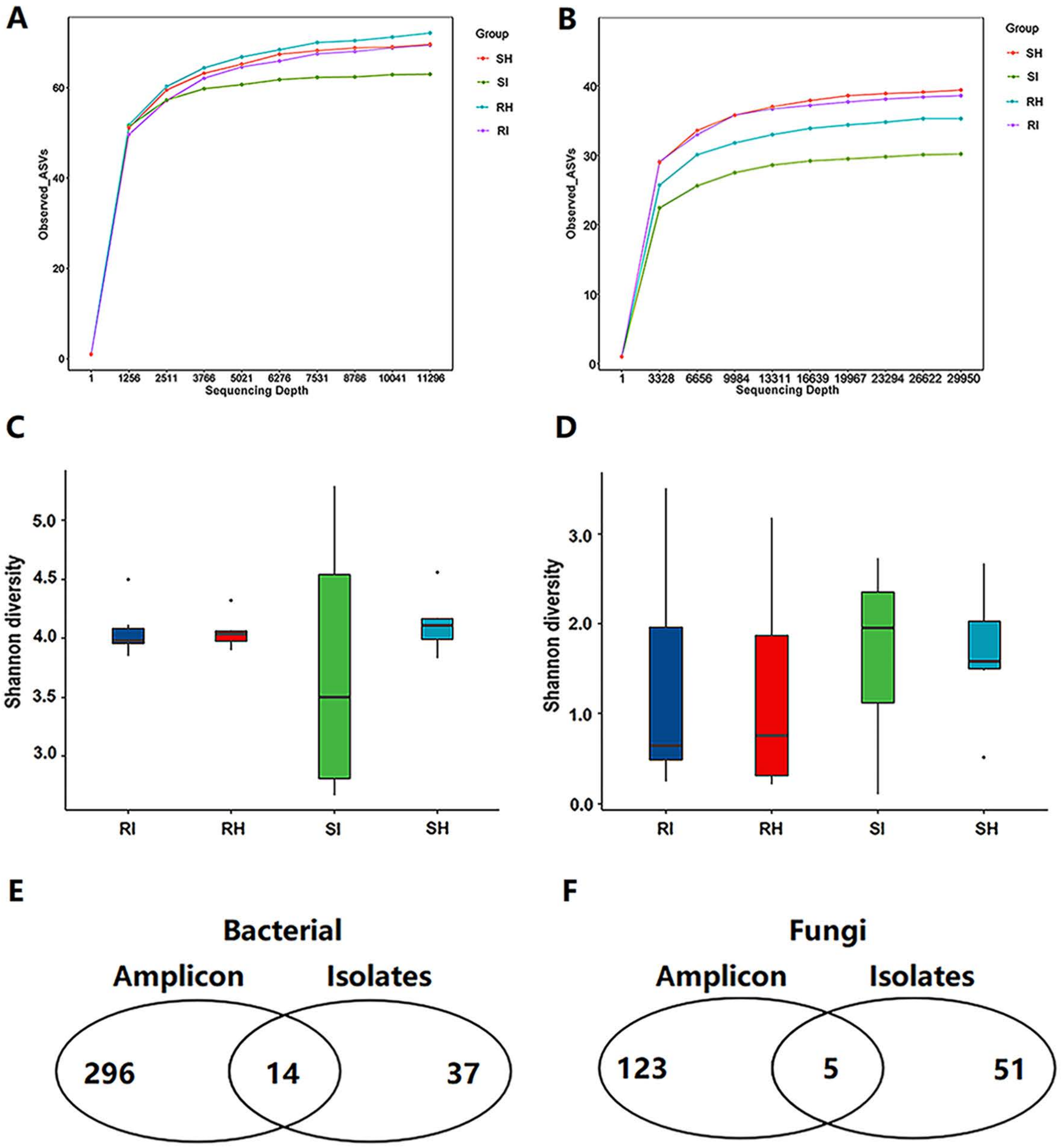

Fig. S2. Sequencing depth of microbial taxa in *T. controversa*-infected and uninfected plants in both resistant and susceptible cultivars. (**A**) Sequencing depth for 16S rRNA (bacteria). (**B**) Sequencing depth for ITS (fungi). (**C**) Shannon diversity analysis of bacterial community composition. (**D**) Shannon diversity analysis of fungal community composition. (**E**) Overlapping bacteria of isolation and amplicon sequencing at the species level. (**F**) Overlapping fungi of isolation and amplicon sequencing at the species level. RI: *T. controversa*-infected resistant cultivar, RH: uninfected resistant cultivar, SI: *T. controversa*-infected susceptible cultivar, SH: uninfected susceptible cultivar.

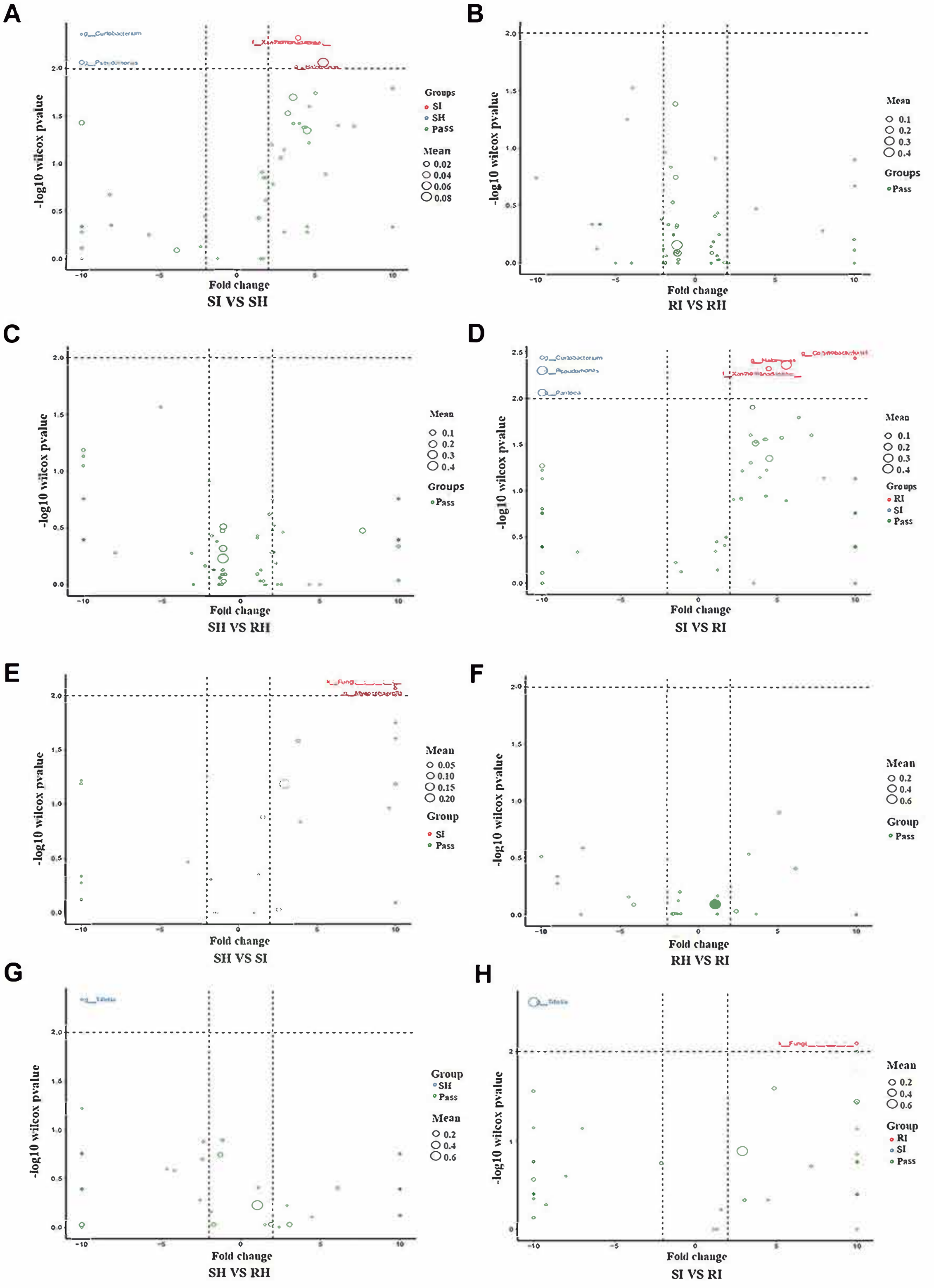

Fig. S3. Differences in abundances of microbes in resistant and susceptible cultivars in *T. controversa*-infected and uninfected groups. (**A**) Differences in abundances of bacterial genera in *T. controversa*-infected (SI) and uninfected susceptible cultivars (SH). (**B**) Differences in abundances of genera in *T. controversa*-infected (SI) and uninfected resistant cultivars (RH). (**C**) Differences in abundances of bacterial genera in uninfected susceptible cultivars (SH) and resistant cultivars (RH). (**D**) Differences in abundances of bacterial genera in *T. controversa*-infected susceptible cultivars (SI) and resistant cultivars (RI). (**E**) Differences in abundances of fungal genera in uninfected (SH) and *T. controversa*-infected susceptible cultivars (SI). (F) Differences in abundances of fungal genera in uninfected (RH) and *T. controversa*-infected resistant cultivars (RI). (**G**) Differences in abundances of fungal genera in uninfected susceptible (SH) and resistant cultivars (RH). (**H**) Differences in abundances of fungal genera in *T. controversa*-infected susceptible (SI) and resistant cultivars (RI). RI: *T. controversa*-infected resistant cultivar, RH: uninfected resistant cultivar, SI: *T. controversa*-infected susceptible cultivar, SH: uninfected susceptible cultivar.

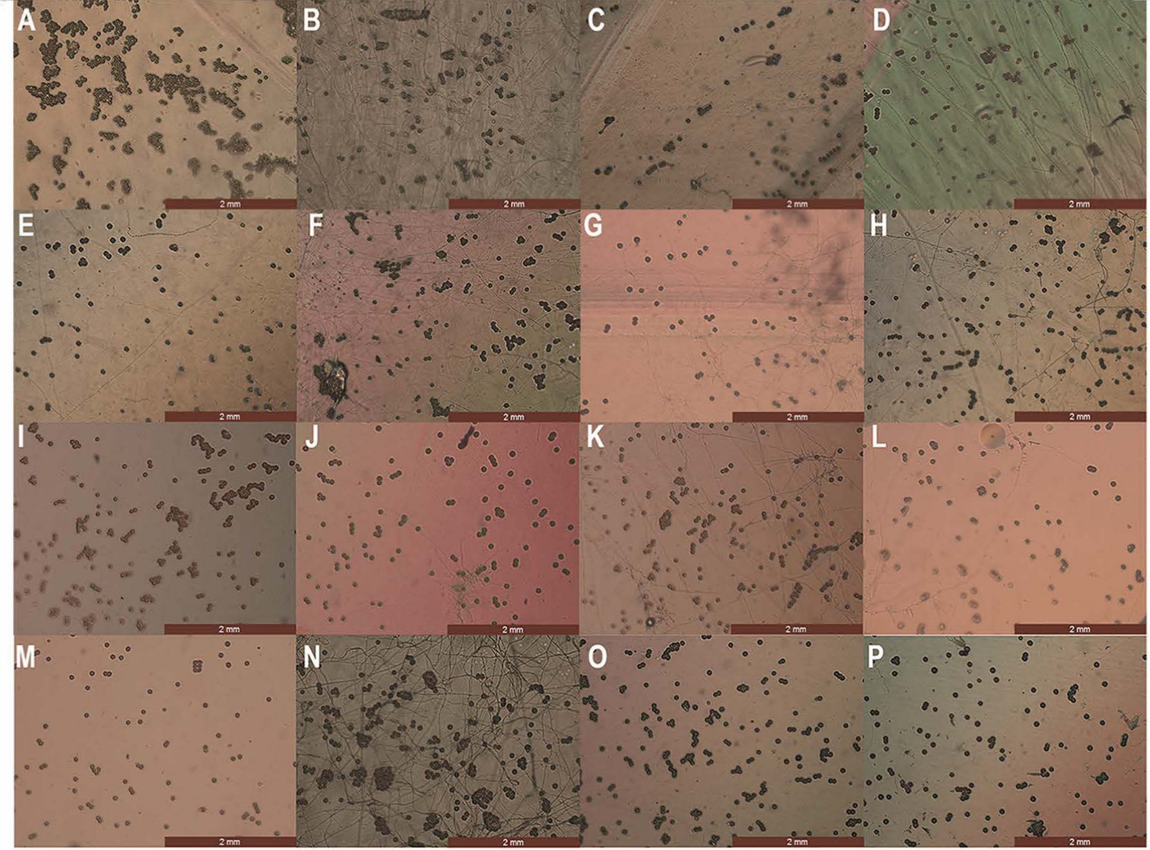

Fig. S4. Potential antagonism from isolation: **(A)** *Leucobacter chromiiresistens*, **(B)** *Curtobacterium flaccumfaciens*, **(C)** *Bacillus nealsonii*, **(D)** *Pantoea brenneri*, **(E)** *Sarocladium* sp., **(F)** *Fusarium proliferatum*, **(g)** *Fusarium fujikuroi*, **(H)** *Fusarium avenaceum*, **(I)** *Aspergillus penicillioides,* **(J)** *Erwinia tasmaniensis*, **(K)** *Alternaria* OTU105, **(I)** *Fusarium verticillioides,* **(M)** *Cladosporium tenellum*, **(N)** *Phoma* 4 BRO-2013, **(O)** *Pseudomonas lurida*, **(P)** Control.

Table S1. Media components for isolation of bacteria and fungi.

Media	Components
LB	Tryptone 10 g/L; Yeast extract 5 g/L; NaCl 10 g/L; Agar 15 g/L.
Per 25 NA	Peptone 1.25 g/L; Beef extract 0.75 g/L; NaCl 1.25 g/L; Agar 15 g/L.
PDA	Potato extract 200 g/L; glucose 20 g/L; Agar 15 g/L.
Per 25 PDA	Potato extract 200 g/L; glucose 20 g/L; Agar 15 g/L.
TSA	Tryptone 15 g/L; Soy papain hydrolyzate 5 g/L; NaCl 5 g/L; Agar 15 g/L.
R2A	Yeast extract powder 0.5 g/L; Peptone 0.5 g/L; Casein Hydrolyzate 0.5 g/L; Glucose 0.5 g/L; Soluble Starch 0.5 g/L; KH_2PO_4 0.3 g/L; $MgSO_4$ 0.024 g/L; $C_3H_3NaO_3$ 0.3 g/L; Agar 15 g/L.
Rice	Rice 300 g/L, Agar 20 g/L.
TWYE	Yeast extract 0. 25 g/L; KH_2 PO_4 0. 5 g/L; Agar 15 g/L.
CMA	Maize flour 5 g/L; Peptone 0.1 g/L; Glucose 1 g/L
DG 18	Casein peptone 5 g/L; Anhydrous dextrose 10 g/L; KH_2PO_4 1 g/L; $MgSO_4$ 0.5 g/L; Chlornitramine 0.002 g/L; Chloramphenicol 0.1 g/L; Agar 15 g/L.
MEA	Malt extract 30 g/L; Soybean peptone 3 g/L, Agar 15 g/L.
RBM	Peptone 5 g/L; Glucose 10 g/L; KH_2PO_4 1 g/L; MgSO4 0.5 g/L; Bengal Red 0.03 g/L; Chloramphenicol 0.1 g/L; Agar 15 g/L.
V8	V-8 Juice 200 g/L $CaCO_3$ g/L; Agar 15 g/L.

Table S5. The total relative abundance of the main endophytic microorganisms obtained by amplicon sequencing.

	Name	RI	RH	SI	SH
Bacterial	*Leucobacter chromiiresistens*	0.0000	0.0000	0.1149	0.0073
	Curtobacterium flaccumfaciens	0.0000	0.0000	0.2891	0.0432
	Curtobacterium plantarum	0.0000	0.0154	0.000	0.6429
	Pantoea anthophila	0.2074	0.0000	0.0000	0.0000
	Pantoea brenneri	0.2631	0.0000	0.1746	0.0086
	Bacillus nealsonii	0.4908	0.9010	0.2658	0.1530
	others	0.0385	0.0834	0.1553	0.1447
Fungi	*Didymella sp.*	0.0000	0.0000	0.0001	0.2684
	Coniothyrium aleuritis	0.0000	0.0000	0.0001	0.1581
	Fusarium sp. P89003	0.0000	0.0000	0.1083	0.0000
	Fusarium fujikuroi	0.0000	0.0000	0.1162	0.0000
	Sarocladium sp.	0.0000	0.0000	0.3193	0.0000
	Fusarium proliferatum	0.0000	0.0000	0.3636	0.0000
	Aureobasidium pullulans	0.8697	0.0000	0.0000	0.2686
	others	0.1302	1.0000	0.0920	0.3047

Note: RI indicated *T. controversa*-infected resistant cultivar, RH indicated uninfected resistant cultivar, SI indicated *T. controversa*-infected susceptible cultivar, SH indicated uninfected susceptible cultivar.

Table S6. PERMANOVA for bacterial and fungal Bray−Curtis analysis.

**UniFrac tests**	**R2-value**	***P*-value**
Bacterial Bray−Curtis	0.39	0.002
Fungi Bray−Curtis	0.41	0.001

Note: *P*-value < 0.05 indicated significant difference.

Table S8. Topological properties of co-occurring networks.

Network parameters	RH, RI, SH	SI
Number of edges	93.00	25.00
Number of positive edges	56.00	16.00
Number of negtive edges	37.00	9.00
Number of nodes	38.00	18.00
Clustering coefficient	0.61	0.48
Network density	0.13	0.16
Connected components	3.00	2.00
Average path length	1.98	2.80
Network diameter	5.00	8.00
Network radius	2.00	1.00
Network centralization	0.09	0.21
Average number of neighbors	4.90	2.78

Note: RI: *T. controversa*-infected resistant cultivar, RH: uninfected resistant cultivar, SI: *T. controversa*-infected susceptible cultivar, SH: uninfected susceptible cultivar.

Table S10. Total relative abundance of potential key taxa identified by culture-dependent and culture-independent pattern against *T. controversa*

Name	RI (%)	RH (%)	SI (%)	SH (%)
Phoma sp. 4 BRO-2013	0.00	0.00	0.41	2.86
Sarocladium sp.	0.00	0.00	31.93	0.00
Pseudomonas lurida	0.11	0.00	0.00	1.37
Pantoea brenneri	26.31	0.00	17.46	0.87
Curtobacterium flaccumfaciens	0.00	0.00	28.92	4.32
Fusarium fujikuroi	0.00	0.00	11.63	0.00
Fusarium proliferatum	0.00	0.00	36.37	0.00
Leucobacter chromiiresistens	0.00	0.00	11.50	0.74
Bacillus nealsonii	49.08	90.11	26.59	15.31
Fusarium avenaceum	0.00	0.00	10.41	0.00
Aspergillus penicillioides	0.00	0.00	2.08	0.00
Alternaria sp.	0.76	2.08	7.91	18.80
Erwinia tasmaniensis	0.00	8.21	0.00	0.00
Cladosporium tenellum	0.00	0.00	7.98	0.00
Fusarium verticillioides	0.00	0.00	1.25	0.00
Curtobacterium plantarum	0.00	1.55	0.00	64.29
Pantoea agglomerans	0.00	0.00	8.53	3.65
Pseudomonas graminis	0.03	0.00	0.59	0.96

Note: RI: *T. controversa*-infected resistant cultivar; RH: uninfected resistant cultivar; SI: *T. controversa*-infected susceptible cultivar; SH: uninfected susceptible cultivar.

Table S11. The suppression of germination rates from the potential antagonist isolates identified with culture-dependent pattern on the germination of teliospores of *T. controversa*

Antagonist isolates	Germination rate (%)	P-value
*Phoma sp. 4 BRO-2013 **	0.25 ± 0.25	0.000000
*Sarocladium sp.**	1.30 ± 0.30	0.000005
*Pseudomonas lurida**	1.55 ± 0.05	0.000000
*Pantoea brenneri**	2.00 ± 0.10	0.000978
*Curtobacterium flaccumfaciens**	2.03 ± 0.48	0.000056
*Fusarium fujikuroi**	2.33 ± 0.58	0.000151
*Fusarium proliferatum**	2.67 ± 0.58	0.000202
*Leucobacter chromiiresistens**	3.00 ± 1.00	0.002278
*Bacillus nealsonii**	3.03 ± 0.38	0.000054
*Fusarium avenaceum**	3.36 ± 1.15	0.003126
*Aspergillus penicillioides**	6.00 ± 0.26	0.000442
Alternaria sp.*	6.90 ± 0.20	0.003285
Erwinia tasmaniensis	6.80 ± 0.87	0.176533
Cladosporium tenellum	6.82 ± 1.23	0.323981
Fusarium verticillioides	7.33 ± 1.20	0.696704
Curtobacterium plantarum	8.67 ± 1.53	0.131777
*Pantoea agglomerans**	13.50 ± 1.50	0.002464
*Pseudomonas graminis **	16.83 ± 2.16	0.001807
Control	7.63 ± 2.85	0.996359

Note: (*) indicates significance difference (*P*-value < 0.05).
